# Mediating and moderating effects of perceived social support on the relationship between discrimination and well-being: A study of South Koreans living in Japan

**DOI:** 10.3389/fpsyg.2022.922201

**Published:** 2022-07-28

**Authors:** Joonha Park, Mohsen Joshanloo

**Affiliations:** ^1^Graduate School of Management, Nagoya University of Commerce and Business, Nagoya, Japan; ^2^Department of Psychology, Keimyung University, Daegu, South Korea

**Keywords:** discrimination, wellbeing, social support, Korean migrants, Japanese society, ingroup inclusion, need to belong, ethnic identity

## Abstract

We examined the relationship between discrimination and mental wellbeing among South Korean residents (*N* = 181) in Japan. The roles of need for belonging (NTB) as a mediator and identification with one’s group as a moderator of this relationship were examined. Perceived social support was also examined as both a potential moderator and mediator. We also included a measure of perceived in-group inclusion in the host society, the Circle of Ingroup Inclusion (CII), to examine its influence on the relationship between discrimination and wellbeing. Three types of coping styles-active constructive coping, passive constructive coping, and destructive coping-were controlled for in the analysis. Results showed that participants’ educational level, socioeconomic status, and different coping styles predicted wellbeing; however, discrimination was the strongest (negative) predictor of wellbeing. Social support was both a moderator and mediator of the relationship between discrimination and wellbeing, suggesting that perceived social support not only buffers the negative effect of discrimination on wellbeing, but also partially explains the negative association between discrimination and wellbeing. NTB was not a significant mediator. Identification with one’s ethnic group and perceived membership in one’s group also did not affect the relationship. The results suggest that it is important to consider social support based on interpersonal relationships among members of minority groups in Japanese society. The psychological factors involved in acculturation processes may be different in different ethnic groups. This study calls for greater consideration of group-specific characteristics in understanding acculturation processes and interactions between groups in society.

## Introduction

### Discrimination in minority groups

People can feel socially excluded in subtle forms in daily lives. This happens more often in ethnic minority groups who have different cultural backgrounds from the majority. The members feel excluded in both their interpersonal relations and at the societal level when they receive messages that themselves as the members of the ethnic group, or their social groups, are devalued and marginalized by mainstream society (Wesselmann et al., 2019). The social exclusion often exists as out-group discrimination in intergroup relations.

Discrimination is a crucial factor that explains mental problems of ethnic minority populations (Williams et al., 1997). Molero et al. (2013) suggests four types of discrimination depending on its visibleness and the target. The study found that subtle (vs. blatant) and individual (vs. group) discrimination showed the most harmful effect on psychological wellbeing of the stigmatized. Many studies so far have exclusively focused on immigration societies (e.g., European countries, United States). Although East Asian societies are known as relatively traditional and homogenous, they are becoming rapidly multicultural with increasing foreign populations in this global age. In awareness of the importance of examining different minority groups across contexts, the current study examines South Korean (Korean, hereafter) newcomers in Japan regarding their psychological experiences and the effects on wellbeing.

### Korean residents in Japan

Koreans are the second largest migrant group in Japan, following the Chinese (Japan Ministry of Justice, 2020). The majority of Korean newcomers comprises high-skilled individuals who settled down for career development and their accompanying families. Lee et al. (2016) identifies interpersonal relationship as one of the major stressors in this group’s acculturation. Dominants generally appear to show less prejudicial attitudes toward high-skilled (vs. low-skilled) immigrants (Hainmueller and Hiscox, 2010). Hence, the discrimination experienced in Korean newcomers can be relatively mild. However, there have been long historical conflicts between Japan and South Korea (Jin et al., 2022). As implied in far-right groups’ hate speech reported on media, there are certain forms of prejudice and discrimination toward this minority group. In fact, inter-group conflicts occur rather implicitly, and subtle experiences influence individuals’ acculturation and wellbeing (Molero et al., 2013). Therefore, the current study aims to examine the perceived level of discrimination in Korean newcomers and how their experiences influence wellbeing.

### Social support and need to belong

In this paper, social support refers to the feelings and perception of being cared for by others and having a reliable network to turn to when needed in daily lives or in times of threat (Taylor, 2011), not instrumental support or social-support-seeking (see also, Pascoe and Smart Richman, 2009). Social support helps individuals maintain wellbeing in stressful environments (Cobo-Rendón et al., 2020). In contexts of social exclusion, support buffers the negative relationship between discrimination and psychological distress (e.g., Ajrouch et al., 2010). Despite some supportive findings, however, Schmitt et al. (2014) meta-analysis suggests that the moderation or buffering effects are inconclusive. Therefore, our current study aims to test the effect with Korean newcomers in Japan. On the other hand, the mediating role of perceived social support is little investigated. Interestingly, Goreis et al. (2020) identifies social support not moderating but mediating between discrimination and psychological distress among Russian immigrants in Germany. The study suggests that those who face discrimination tend to perceive social support less available, which leads to increase in distress. To relate it to the current study, it is expected that those who experience more discrimination would perceive support less available, which leads to decrease in wellbeing.

NTB is another potential mediator in the relationship. This concept refers to a human emotional need to affiliate with and be accepted by members of a group (Leary, 2010). In an experimental study, socially excluded individuals experienced not only higher (lower) negative (positive) emotions but also higher NTB (Chen et al., 2017). This phenomenon can be interpreted with *threatened belonging* (Baumeister and Leary, 1995). According to the need-threat theory of ostracism, one’s sense of belongingness is basic human need; thus, it is an important part of one’s wellbeing. However, rejection-related experiences, such as ethnic discrimination in the host society, would threaten the basic need. Thus, the individual would increase NTB to fill the lack. Thus, higher NTB would reflect a lower sense of belongingness at present. If the deficiency goes on for a long time, because of lack of supportive environments or because of rejection, various long-term negative effects will follow (Richman and Leary, 2009). Based on this reasoning, it is expected that NTB would mediate the relationship between discrimination and wellbeing.

### Ingroup identification

According to social identity theory, one’s ingroup identification is an important part of self-concept (Tajfel et al., 1979). Positive ingroup membership internalized in self can prevent the individual from out-group threat, which can reduce the negative effects on mental health. Ingroup identification also fosters individuals to believe in their capacity to cope with the negative experience, which contributes to wellbeing (Outten et al., 2008). Accordingly, studies of intergroup conflicts support that ingroup identification moderates the negative effects of discrimination on wellbeing (e.g., Heim et al., 2010; Lee and Ahn, 2013). However, this moderation is inconclusive. For example, some studies report no supportive findings with Russian immigrants in Germany (Goreis et al., 2020) or Muslim women in New Zealand (Jasperse et al., 2012). To our knowledge, however, the relationships have been rarely examined East Asian minorities. Therefore, the current study aims to fill this gap by testing whether the moderation effect of ingroup identity is supported in South Koreans in Japan.

We speculate that the perceived inclusion of the ethnic ingroup in the host society can moderate the relationship between discrimination and wellbeing. Whereas ethnic discrimination reflects a specific form of exclusion, one can hold a sense of ingroup belongingness in terms of to what extent the ethnic ingroup is included and respected by the host group in the larger society. In relation to acculturation theory, this concept can be understood as ethnic group members’ theories of how the host group expect the minority group to acculturate to the larger society in terms of maintenance of the heritage culture and interactions with the host group, Berry, 2017). It is possible that inclusion (or exclusion) of the ethnic ingroup also influences wellbeing and acculturation in the members of the minority group. Thus, we explored whether the sense of inclusion of the ethnic ingroup moderates the relationship between discrimination and wellbeing.

### The present study

As an initial approach to understand the dynamic process of intergroup relations in Japanese society, we tested the following hypotheses with Korean residents.

H1. Perceived discrimination in the larger society would predict overall aspects of wellbeing negatively. We explored whether the negative association would be significant, regardless of personal coping strategies and other demographic factors.
H2-1. Perceived social support would reduce the negative association between discrimination and welleing.
H2-2. Perceived social support would mediate the relationship between discrimination and wellbeing.
H3. NTB would mediate the relationship between discrimination and wellbeing. Discrimination would influence wellbeing negatively partly through the increased NTB.
H4. Ethnic identification would reduce the association between discrimination and wellbeing.

We additionally explored whether perceived ingroup inclusion in the host society would function positively by protecting wellbeing from discrimination.

## Methods

### Participants

A total of 181 South Korean residents in Japan (female 47.5%, mean age = 36.06, *SD* = 8.751) were recruited through a Korean community webpage on Facebook in February, 2022. 63.5% answered that they came to Japan for work, 17.1% for study, 16.0% for marriage, and 3.3% for other purposes. The highest education level for 88.8% of participants was undergraduate or higher categories. The average length of stay in Japan was 9.3 years (*SD* = 7.3 years).

### Measures

#### Discrimination

We measured ethnic discrimination with two scales: Perceived Discrimination Scale (PDS, 5 items, Berry, 2017) and Everyday Discrimination Scale (EDS, 5 items, Sternthal et al., 2011). Each item of the PDS was rated on a 5-point scale ranging from 1 = *strongly disagree* to 5 = *strongly agree* (e.g., “*I have been teased or insulted because of my Korean background*”). Each item in the EDS was rated on a 6-point scale ranging from 1 = *never* to 6 = *almost everyday* (e.g., “*You receive poorer service than other people at restaurants or stores*”). Because the two measurements are rated on different units [i.e., the level of (dis)agreement for PDS, and the level of frequency for EDS], we did not unify the scales and used the original 5 and 6-point scales, respectively. The scree plot for the 10 items showed that a one-factor structure would be the best choice for this scale (eigenvalue = 4.396), with factor loadings ranging between 0.582 and 0.768.

#### Wellbeing

The study included four wellbeing variables. Internationally Reliable Short-Form of the Positive and Negative Affect Schedule (I-PANAS-SF, 10 items, Thompson, 2007) measured emotional experiences for the past week. Satisfaction With Life Scale (SWLS, 5 items, Diener et al., 1985) measured overall life satisfaction. Psychological wellbeing (PWB, 18 items, Ryff et al., 2010) measured six aspects of life: autonomy, environmental mastery, personal growth, positive relations with others, purpose in life, and self-acceptance. Each item in all these scales was rated on a 5-point scale. Instead of a linear composite (e.g., averaging the four variables), we used principal axis factoring to measure wellbeing as a latent construct and to capture the common variance among the four variables. The resulting factor scores serve as a general wellbeing variable (Joshanloo, 2021). A factor analysis with a single factor was conducted, and factor scores were saved for all participants. The resulting variable was used as an outcome in this study (eigenvalue = 1.705, range of factor loadings = 0.858 and 0.332, for psychological wellbeing and positive affect, respectively).

#### Social support

Enriched Social Support Inventory (ESSI, Mitchell et al., 2003) consists of 7 items; however, we selected 6 items measuring perceived availability of social support excluding an item asking marriage status (Goreis et al., 2020). The scale asks about the availability of anybody regardless of ethnic groups who can help around the individual, not specific others like partners or friends (e.g., “*Is there someone available to give you good advice about a problem?*”). Each item was rated on a 5-point scale (1 = *none of the time* to 5 = *all the time*).

#### Need for belonging

We used a single-item scale of NTB (SIN-B, Nichols and Webster, 2013, *“I have a strong need to belong”*), rated on a 5-point scale. This measurement showed good test-retest reliability across 4 months and validity of this measurement in the previous study.

#### Coping styles

Brief COPE (Carver, 1997) was adopted and revised to measure discrimination-specific coping (Goreis et al., 2020). Following the previous study, we adopted 24 items out of the original 28 items that include emotional and instrumental aspects of support. Each item was rated on a 6-point scale (1 = *strongly disagree* to 5 = *strongly agree*). We factor-analyzed 12 constructive coping and 12 destructive coping items separately using principal axis factoring and promax rotation. We used only items with factor loadings above 0.3 to calculate new variables and dropped items with lower loadings. The scree plot for the constructive coping items indicated that a two-factor structure (eigenvalues = 2.727 and 1.846) would be best. Four items loaded on the first factor (factor loadings ranged between 0.772 and 0.866.) This factor was labeled *active constructive coping* (e.g., “*I try to come up with a strategy about what to do*”). Six items loaded on the second factor (between 0.375 and 0.674) labeled *passive constructive coping* (e.g., “*I look for something good in what is happening*”). Two items did not have sufficiently high loadings and were excluded. The scree plot for 9 negative items indicated a single factor structure (eigenvalue = 3.228, factor loadings ranged from 0.411 to 0.725). This factor was labeled *destructive coping* (e.g., “*I’ve been criticizing myself*”). Three items with low loadings were excluded.

#### Ethnic identity

Three items were adopted from the Ethnic Identification Scale (EIS, Berry, 2017). Each item was rated on a 5-point scale (1 = *strongly disagree* to 5 = *strongly agree*, e.g., “*I am proud of being Korean*”).

#### Circle of ingroup inclusion

Perceived ingroup inclusion was developed following the measure of the Inclusion of Ingroup in the Self (Tropp and Wright, 2001). The circle of ingroup inclusion (CII) measures perception of the inclusion of the ethnic ingroup in the larger society by varying the difference between the two groups. Two circles, one reflecting ethnic (Korean) ingroup and the other reflecting Japanese group, were prepared. Participants were asked to select one of the seven patterns that vary in the distance between the two circles, so that a greater score reflects a higher level of inclusion of the ethnic ingroup. This measure showed negative correlations with PDS (*r* = –0.375, *p* < 0.001) and EDS (*r* = –0.186, *p* = 0.012), but no relationship with EIS (*r* = 0.013, *p* = 0.865). That is, the more the ingroup perceives being excluded from the larger society, the more discrimination is experienced, independent of ingroup identification. These support construct validity and discriminant validity of the new measure.

#### Demographic information

Age, gender, education level, socioeconomic status (5-point scale), length of stay in Japan, and purpose of stay were included at the end of the questionnaire.

### Procedure

The study was conducted as a part of a project on the childcare and wellbeing in Asian societies. Invitation emails were sent to those who showed an interest in the survey. After reading the consent form, participants completed the survey which took 15∼20 min. The measures for our main variables were followed by other irrelevant measures to the current study (e.g., childcare experiences and gender role beliefs). After completion, participants were reimbursed with a 1,000 yen voucher. The study was approved by the Research Ethics committee of the first author’s university (no. 21067).

## Results

### Overview

All of the main variables are listed in Table 1. To see the mean values, participants’ ethnic discrimination experiences appeared to be mild (below the mid-point). Overall, the ratings on other variables were also positive, consistent with the mild circumstances observed in the high-skilled Koreans (Lee et al., 2016).

**TABLE 1:** List of the main variables in the current study and their reliabilities (α), means and *SD*s.

Measures	Cronbach’s α	Mean	*SD*
Discrimination	0.880	1.950	0.725
Wellbeing	0.663	0.000	0.913
PANAS_negative	0.821	2.065	0.836
PANAS_positive	0.681	3.069	0.778
SWLS	0.859	3.263	0.851
PWB	0.810	3.648	0.513
Active constructive coping	0.883	3.711	0.935
Passive constructive coping	0.712	2.846	0.834
Destructive coping	0.816	2.082	0.735
CII	–	3.980	1.509
Ethnic identity	0.798	3.900	0.848
Social support	0.920	4.059	0.878
NTB	–	3.350	1.129

### Discrimination as a predictor of wellbeing

In model 1, wellbeing was regressed on discrimination *R*^2^ = 0.260, *F*(1, 179) = 62.752, *p* < 0.001. As shown in Table 2, discrimination was a significant predictor. In model 2, we controlled for the demographic variables of the study, and again, discrimination was a significant predictor *R*^2^ = 0.399, *F*(6, 169) = 18.665, *p* < 0.001. In a third model, we controlled for the three coping variables, *R*^2^ = 0.524, *F*(9, 166) = 20.325, *p* < 0.001. Table 2 shows that discrimination was still a significant predictor of wellbeing, after holding all the demographic and coping variables constant. Thus, the findings give strong support to H1.

**TABLE 2:** Serial models of discrimination predicting general wellbeing.

Predictor	B	CI: Lower	CI: Upper	*t*	*p*	β
**Model 1**						
Discrimination	**–0.642**	–0.802	–0.482	–7.922	0.000	–0.509
**Model 2**						
Discrimination	**–0.630**	–0.779	–0.482	–8.389	0.000	–0.503
Male	**–0.285**	–0.510	–0.061	–2.507	0.013	–0.157
Age	0.001	–0.016	0.019	0.153	0.879	0.013
Education	**0.184**	0.049	0.320	2.685	0.008	0.166
SES	**0.207**	0.082	0.333	3.254	0.001	0.202
Length of stay	0.000	–0.002	0.002	0.007	0.994	0.001
**Model 3**						
Discrimination	**–0.483**	–0.624	–0.342	–6.775	0.000	–0.385
Male	–0.199	–0.402	0.004	–1.933	0.055	–0.110
Age	–0.007	–0.023	0.009	–0.805	0.422	–0.063
Education	**0.175**	0.053	0.297	2.834	0.005	0.158
SES	**0.188**	0.073	0.303	3.234	0.001	0.183
Length of stay	0.000	–0.002	0.002	–0.027	0.978	–0.002
Active constructive coping	**0.166**	0.055	0.276	2.968	0.003	0.169
Passive constructive coping	**0.169**	0.048	0.290	2.763	0.006	0.157
Destructive coping	**–0.399**	–0.549	–0.249	–5.245	0.000	–0.318

*CI, confidence interval. Bold values denote statistical significance at the p < 0.05 level.*

### Social support as a moderator

**FIGURE 1:**
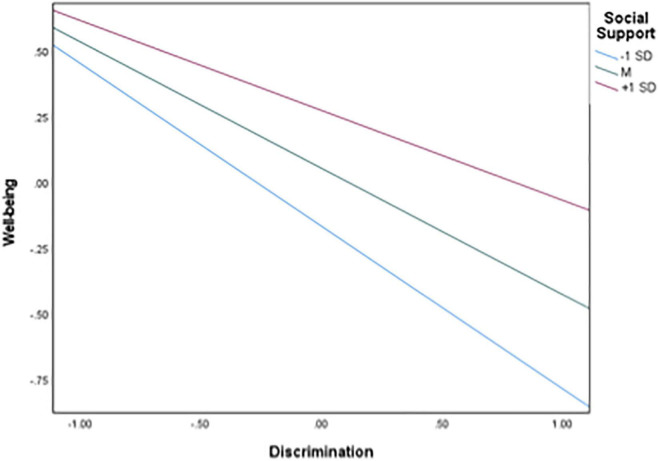
Results of the simple slope analysis.

A moderation analysis with social support was performed, controlling for gender, age, education, socio-economic status, and length of stay, *R*^2^ = 0.470, *F*(8, 167) = 18.499, *p* < 0.001. In support of H2-1, social support was a significant moderator (interaction term coefficient = 0.156, *p* = 0.035). The interaction is shown in Figure 1. As can be seen, the negative relationship between discrimination and wellbeing is weaker at higher levels of social support, whereas this relationship is stronger at lower levels of social support.

### Mediation analysis

In a mediation analysis using the process macro (Hayes, 2022), we examined whether social support and NTB are two mediators of the relationship between discrimination and wellbeing, controlling for the same demographic variables used in the regression analyses. The number of bootstrap samples for percentile bootstrap confidence intervals was 5,000. Discrimination was a significant predictor of social support (*b* = –0.417, *p* < 0.001) and need to belong (*b* = –0.235, *p* = 0.045). Social support (but not NTB) was a significant predictor of wellbeing (*b* = 0.266, *p* < 0.001), in support of H2-2 but not H3. The bootstrap confidence interval of the indirect effect of discrimination via social support did not include zero (–0.204 and –0.041), whereas the indirect effect via NTB included zero (–0.026 and 0.035). Hence, only support is a significant mediator. Considering that the direct effect of discrimination on wellbeing remained significant (*b* = –0.521, *p* < 0.001), it can be concluded that support partially mediated the relationship.

### Ingroup identification or perceived ingroup inclusion as moderators

Using the process macro, a moderation analysis with centered variables was performed, *R*^2^ = 0.461, *F*(10, 165) = 14.128, *p* < 0.001 (Table 3). In the model, discrimination negatively, and ingroup identification, education, and socioeconomic status positively predicted wellbeing. None of the interaction terms were significant. Hence, the moderation effect of ethnic identification (H4) was not supported in our study. Also, we did not find evidence that perceived ingroup inclusion in the host society influences the association between individual-level discrimination and wellbeing.

**TABLE 3:** Moderation analysis on wellbeing in South Korean residents.

Predictor	Unstandardized coefficient	CI: Lower	CI: Upper	*t*	*p*
Discrimination	**–0.563**	–0.714	–0.412	–7.349	0.000
Ingroup identification	**0.270**	0.145	0.396	4.249	0.000
Discrimination*ingroup identification	0.031	–0.125	0.188	0.393	0.695
CII	0.046	–0.028	0.121	1.227	0.222
Discrimination*CII	0.028	–0.060	0.115	0.624	0.533
Male	–0.193	–0.412	0.027	–1.733	0.085
Age	–0.002	–0.019	0.015	–0.206	0.837
Education	**0.196**	0.065	0.327	2.948	0.004
SES	**0.213**	0.090	0.336	3.415	0.001
Length of stay	0.000	–0.002	0.002	–0.079	0.937

*CI, confidence interval. Bold values denote statistical significance at the p < 0.05 level.*

## Discussion

The current study supports negative associations between perceived and experienced ethnic discrimination and wellbeing in Korean newcomers in Japan. Although education level, socioeconomic status, and adaptiveness of coping strategies were predictors of wellbeing, discrimination was still significant even when those factors were controlled for. In the serial analyses, discrimination was a stronger predictor of wellbeing than other variables included, even than socioeconomic status. In literature, most studies have focused on psychological malfunctioning and distress, especially in minority groups in societies where discrimination is more visible (e.g., African Americans, Williams et al., 1997). However, our findings suggest that the effects can be also applied to the general aspect of wellbeing. Those who experience greater social exclusion experience not only more negative affect and less positive affect but also lower life satisfaction and psychological wellbeing (autonomy, environmental mastery, personal growth, positive interpersonal relations, purpose in life, and self-acceptance). Moreover, the current study implies that the negative association exists even in minority groups of high status newcomers whose discrimination experiences are relatively mild (Hainmueller and Hiscox, 2010).

In support of the buffering role, the current study identified the moderation effect of perceived social support on the relationship between discrimination and wellbeing in the Korean sample. It also found supporting evidence of the mediation effect, implying that social support (i.e., perceived availability of someone who can help when needed) partly explains the association between discrimination and wellbeing. These findings suggest the importance of the perceived social support for wellbeing in ethnic minority groups in the larger society. In particular, the mediation model is relatively new in the study of ethnic discrimination. Loneliness may be an important concept to explain the association. Discrimination is associated with feelings of loneliness, especially in minority groups such as older retirees (Lee and Bierman, 2019) and people with psychotic disorders (Świtaj et al., 2015). Loneliness is suggested to have an important impact mental health (Wang et al., 2020). Studies also suggest large negative correlations between loneliness and social support—lonely people are less likely to perceive availability of social support (for review, Wang et al., 2018). Given the negative impact of loneliness on mental health, our findings suggest the study of loneliness in ethnocultural minorities as an important future direction in acculturation research.

In that social support is a crucial factor for mental health of socially marginalized individuals (Cobo-Rendón et al., 2020), the current findings may provide implications for how to support ethnic minority groups for their positive acculturation and wellbeing. Although active support-seeking behaviors or active provision of support are suggested to be beneficial for reducing the negative effects of ethnic discrimination (Ajrouch et al., 2010), East Asians are less likely to attempt support-seeking behaviors than Westerners, because of concern about interpersonal relationships and face (Taylor et al., 2004). The current findings may reflect the cultural obstacles in support-seeking or receiving in Asian minorities. This is noteworthy in that relationship is a key factor affecting acculturation and wellbeing in Korean newcomers in Japan (Lee et al., 2016). Understanding social norms and cultural practices in Asian minority groups may help develop effective ways of social support in the larger society. Moreover, social support and support-seeking behaviors in Korean and other Asian minority groups in Japanese society are important issues future research needs to keep uncovering.

NTB was not a significant mediator in the current study. There are a few alternative interpretations. First, it might be premature to conclude about the relationship between discrimination and NTB. Indeed, some propose negative associations between perceived discrimination and NTB—because of self-serving bias, individuals with stronger NTB can perceive their own discrimination relatively less than their ingroup fellows’ (Carvallo and Pelham, 2006). The current study did not compare perceived discrimination between the self and the other ingroup members, which calls for future investigation. The other alternative considers a possible group-specific characteristics regarding the NTB-related mechanism. Both belongingness and social support are closely related to social bonding. However, belongingness is oriented toward one’s sense of *group affiliation* more strongly than social support is (Taylor, 2011). Thus, we suspect that, to many Korean newcomers who hold high agency and self-esteem (Lee et al., 2016), the group-oriented need is not so important for coping. In other words, belongingness is not a critical factor in ethnic groups where discrimination-related experiences are mild or the personal agency is high.

Inconsistent with our prediction, ethnic identity did not moderate the association between discrimination and wellbeing. Also, perception of ingroup inclusion in the host society showed no significant effects. These results do not support the buffering effects of one’s ingroup identification or sense of inclusion. They rather imply that the negative effect of discrimination on wellbeing exists regardless of ingroup identification. Effects of ingroup identification may be inconclusive and vary across contexts (Jasperse et al., 2012; Goreis et al., 2020). Although tentative, it is suggested that the functional aspect of the ethnic identity suggested in some contexts (e.g., Heim et al., 2010; Lee and Ahn, 2013) might have not developed enough in newcomers, so that the benefit of ingroup identification is little for their self-regulation against outgroup threats.

This study did have some limitations that need to be addressed in future studies. First, due to the nature of the cross-sectional study that used self-report measures only, we could not demonstrate causal relationships or temporal ordering issues between variables, such as discrimination leading to decrease in perceived availability of social support, or increase in NTB (c.f., Chen et al., 2017). Conducting more controlled experiments or longitudinal studies can get benefit in understanding causal relationships by examining the effects of discrimination-experienced experiences and presence of social support. Also, employing a multiple-item NTB scale can help clarifying the effect of the NTB. Finally, we recommend examining experiences in diverse ethnic minorities including more prejudiced old-comers who may differ in demographic characteristics (e.g., education, income) and ethnic identity to capture a more inclusive picture of the minority groups’ intergroup experiences in the larger society and their effects on wellbeing.

## Conclusion

The current study identified that ethnic discrimination experienced in Korean newcomers living in Japan is negatively associated with general aspects of wellbeing. This finding supports that the negative effects of discrimination are significant even in mildly discriminated groups in society. Perceived availability of social support buffers the negative effects of discrimination on wellbeing. It also mediates the association, implying the importance of interpersonal relationships and connectedness for the wellbeing in the minority group members. Although ethnic identity is positively associated with wellbeing, it does not seem to buffer the negative influence of discrimination on people’s life. Likewise, we did not find the mediating effects of NTB. These findings suggest possible variations in factors affecting acculturation strategies and wellbeing in ethnic minority groups, depending on the levels of ethnic identity, social status, and agency. Future research can benefit by examining diverse ethnic groups to understand the dynamic processes and psychological consequences on wellbeing.

## Data availability statement

The raw data supporting the conclusions of this article will be made available by the authors, without undue reservation.

## Ethics statement

The studies involving human participants were reviewed and approved by Nagoya University of Commerce and Business. The patients/participants provided their written informed consent to participate in this study.

## Author contributions

JP designed the study, developed the conceptual framework, collected the data, and wrote up the manuscript. MJ developed the analytical models, analyzed the data, and summarized the results. Both authors contributed to the article and approved the submitted version.
